# Editorial: Probiotic bacteria-derived effector molecules and their impact on the host in health and disease

**DOI:** 10.3389/fmicb.2022.1089461

**Published:** 2022-11-29

**Authors:** Sabina Górska, Martin Schwarzer, Irma Schabussova, Anna Magdalena Zawilak-Pawlik, Corine Sandström

**Affiliations:** ^1^Laboratory of Microbiome Immunobiology, Hirszfeld Institute of Immunology and Experimental Therapy, Polish Academy of Sciences, Wroclaw, Poland; ^2^Laboratory of Gnotobiology, Institute of Microbiology of the Czech Academy of Sciences, Prague, Czechia; ^3^Institute of Specific Prophylaxis and Tropical Medicine, Medical University of Vienna, Vienna, Austria; ^4^Laboratory of Molecular Biology of Microorganisms, Hirszfeld Institute of Immunology and Experimental Therapy, Polish Academy of Sciences, Wrocław, Poland; ^5^Department of Molecular Sciences, Swedish University of Agricultural Sciences, Uppsala, Sweden

**Keywords:** *Bifidobacterium*, *Lactobacillus*, probiotics, post-biotics, vesicles, exopolysaccharides, peptidoglycan

Bacteria are one of the main modulators of the physiology and the immune system of the host organism (Tlaskalova-Hogenova et al., 2011). Changes in the composition and abundance of bacterial communities, especially, but not exclusively, in the gastrointestinal tract are believed to be a key factor in the host's susceptibility to various diseases (e.g., allergy, inflammatory bowel disease, irritable bowel syndrome, or necrotizing enterocolitis). It is not surprising that prophylactic/therapeutic approaches to modulate the microbiota composition, for example by probiotic bacteria, are being pursued. Along these lines, the history and diversity of probiotics, as well as outlines of conventional *in vitro* assays and *in vivo* models, have been in-depth reviewed by Milner et al. Despite the interest in probiotics as food or supplement use in the clinic, there are huge discrepancies observed in the outcomes of such studies. Duboux et al. suggest that these differences, besides being attributed to variations in the bacterial species and clinical trial protocols, target population, probiotic dosage, or outcome parameters measured, may stem from the methods used to produce the live bioactive ingredients. In their review, the authors suggest that the implementation of molecular level quality controls based on validated probiotic niche factors and effector molecules could improve the functional reliability of probiotic products.

Experimental and clinical data indicate that the efficacy of probiotics differs from strain to strain and therefore, in the screening for new probiotic bacterial products, it is necessary to compare and analyze the characteristics and safety at the strain level. The members of *Enterococcus*, the symbiotic bacteria in the intestine, exhibit dual characteristics of probiotic function and potential pathogenicity. Zhou et al. characterized *Enterococcus durans* A8-1 isolated from a fecal sample of a healthy Chinese infant. Strain A8-1 was able to tolerate and survive the simulated gastric and intestinal juices and showed the potential to colonize the intestinal epithelial cells and competitively exclude enteropathogens with the ability to downregulate the levels of inflammatory cytokines. Zebrafish model was used by Huang et al. to screen forty probiotic bacterial strains for the prevention of inflammatory bowel disease. One candidate, *Bacillus smithii* XY1, restored the intestinal epithelial cell integrity after dextran sodium sulfate (DSS)-induced damage, as well as regulated the expression of inflammation-related genes. Subclinical enteritis poses a significant threat to the chicken industry, severely hampering the growth performance of broilers. Wang et al. showed that treatment of broiler chicks with *Bacillus subtilis* DSM29784 improved the composition and metabolism of the intestinal microbiota, and the intestinal structure, and reduced inflammation and apoptosis in *Clostridium perfringens*-induced intestinal inflammation resulting in improved growth performance.

Probiotics can act at different levels of the host immune system e.g., physical barrier, innate immunity, and adaptive immunity. Kim et al. demonstrated that *Bifidobacterium animalis* ssp. *lactic* HY8002 enhanced the intestinal epithelial cells' barrier integrity by restoring the expression of tight junction proteins, kanamycin-induced reduction in Peyer's patch cell number, and serum and fecal IgA levels in the mouse small intestine. Next, Won et al. studied the effect of oral administration of *Latilactobacillus sakei* ADM14 on obesity and fatty liver in a high-fat diet mouse model. Authors have demonstrated the regulatory activity of strain ADM14 on host lipid metabolism and composition of fecal microbiota that led to decreased body weight gain, fat tissue mass, and liver weight in treated mice. Lee et al. showed that administration of *Lactobacillus rhamnosus* TWK10 enhanced muscle strength in young mice and prevented loss of muscle strength or bone mass in aged mice. Furthermore, learning and memory abilities were improved. Administration of TWK10 had also an influence on gut microbial composition decreasing the aging-associated accumulation of pathogenic bacterial taxa and increasing bacteria-producing short-chain fatty acids (SCFA). Lin et al. compared the effect of dietary fiber supplementation vs. butyrate/probiotic supplementation on semen quality and intestinal microbiome in a boar model. The findings of this study indicate that dietary fiber supplementation improves gut microbiota and promotes SCFA production, which is linked to enhanced spermatogenesis and semen quality. Moreover, the effects of dietary fiber were superior to those of derived metabolites and probiotic supplementation. Finally, Kang and Cai commented on the ability of *Lactobacillus plantarum* GUANKE to boost severe acute respiratory syndrome coronavirus 2 (SARS-CoV-2)-vaccine-induced effective memory immune response by enhancing interferon signaling and suppressing apoptotic and inflammatory pathways. They suggest that using probiotics to boost vaccine efficacy is an inspiration for future research directions.

Recent research indicates that live and proliferating bacteria are not the prerequisite for obtaining health-promoting effects. In experimental studies, the beneficial effects are also achieved by inactivated bacteria or bacteria-secreted extracellular vesicles or certain effector molecules located on the bacterial surface (e.g., peptidoglycan and polysaccharides), secreted by bacteria (e.g., antibacterial peptides) or released after bacterial lysis (e.g., proteins, exopolysaccharides, and DNA) (Lebeer et al., 2018; Pyclik et al., 2020). In this regard, some studies have demonstrated the potential of inactivated microbes. Jhong et al. evaluated the antiosteoporotic effects of heat-killed *Lacticaseibacillus paracasei* GMNL-653 in ovariectomized (OVX) mice. The GMNL-653 exerts anti-inflammatory activity which restored gut microbiota dysbiosis and maintained intestinal barrier integrity in the OVX mice. However, Pyclik et al. showed that both live and heat-killed *Bifidobacterium longum ssp*. *longum* CCM 7952 are able to alleviate the allergy symptomes in mouse OVA-induced allergy model, albeit in a different manner. Studies performed by Pyclik et al. indicated that research on bacterial effector molecules is warranted to elucidate the mechanism of beneficial effects of probiotics.

The effector molecules presented on the bacterial surface, including cell components or metabolic products secreted into the environment, may impart an array of health-promoting properties. One of them are exopolysaccharides (EPS). The EPS have received a lot of attention due to their industrial and therapeutic applications. Brdarić et al. investigated the capacity of eight EPS-producing lactobacilli to adsorb Cd, one of the most significant toxic elements. The most promising EPS turned out to be produced by strain BGAN8 which exhibited a high Cd-binding capacity and prevented Cd-mediated toxicity in intestinal epithelial Caco-2 cells. Khalil et al. isolated and characterized EPS from different Lactic Acid Bacteria and *Bifidobacterium* strains in terms of their antioxidant, antitumor, and periodontal regeneration properties. The antioxidant capacity of EPS varied significantly among tested strains indicating that certain chemical structures provide a beneficial effect. The EPS_5_, composed mainly of galactose, showed the highest cytotoxicity against human cancer lines. Moreover, EPS_5_ treatment selectively regulated the expression of some apoptotic genes expression. Recently, other probiotic molecules i.e., extracellular vesicles have fascinated many scientific groups. Rubio et al. reviewed the EVs derived from Gram-positive and Gram-negative probiotic bacteria in interaction with the host. Novel EV-based technologies are promising for the design of therapies and/or vaccines against infections.

With this topic, we have assembled a set of 15 research articles that bring new insights into active probiotic and post-biotic research areas. These studies highlighted the need for rigorous probiotic selection and the exciting possibility of using inactivated probiotics or bacteria-derived molecules to confer health benefits to the host.

## Author contributions

All authors listed have made a substantial, direct, and intellectual contribution to the work and approved it for publication.

## Funding

This work was supported by grant 19-02261S of the Czech Science Foundation and 8J20AT011 of the Ministry of Education, Youth and Sports of the Czech Republic. This work was supported by grant co-founded by the National Science Centre of Poland under grant decision number UMO-2017/26/E/NZ7/01202 and by the Polish National Agency for Academic Exchange under grant decision number PPN/BIL/2018/1/00005. IS: OEAD PL 03/2022 and CZ 06/2020 and Danube Allergy Research Cluster.

## Conflict of interest

The authors declare that the research was conducted in the absence of any commercial or financial relationships that could be construed as a potential conflict of interest.

## Publisher's note

All claims expressed in this article are solely those of the authors and do not necessarily represent those of their affiliated organizations, or those of the publisher, the editors and the reviewers. Any product that may be evaluated in this article, or claim that may be made by its manufacturer, is not guaranteed or endorsed by the publisher.
